# Molecular and Clinical Studies in 138 Japanese Patients with Silver-Russell Syndrome

**DOI:** 10.1371/journal.pone.0060105

**Published:** 2013-03-22

**Authors:** Tomoko Fuke, Seiji Mizuno, Toshiro Nagai, Tomonobu Hasegawa, Reiko Horikawa, Yoko Miyoshi, Koji Muroya, Tatsuro Kondoh, Chikahiko Numakura, Seiji Sato, Kazuhiko Nakabayashi, Chiharu Tayama, Kenichiro Hata, Shinichiro Sano, Keiko Matsubara, Masayo Kagami, Kazuki Yamazawa, Tsutomu Ogata

**Affiliations:** 1 Department of Molecular Endocrinology, National Research Institute for Child Health and Development, Tokyo, Japan; 2 Department of Pediatrics, Keio University School of Medicine, Tokyo, Japan; 3 Department of Pediatrics, Central Hospital, Aichi Human Service Center, Aichi, Japan; 4 Department of Pediatrics, Dokkyo Medical University Koshigaya Hospital, Saitama, Japan; 5 Division of Endocrinology and Metabolism, National Center for Child Health and Development, Tokyo, Japan; 6 Department of Pediatrics, Osaka University Graduate School of Medicine, Suita, Japan; 7 Department of Endocrinology and Metabolism, Kanagawa Children's Medical Center, Kanagawa, Japan; 8 Division of Developmental Disability, Misakaenosono Mutsumi Developmental, Medical, and Welfare Center, Isahaya, Japan; 9 Department of Pediatrics, Yamagata University School of Medicine, Yamagata, Japan; 10 Department of Pediatrics, Saitama Municipal Hospital, Saitama, Japan; 11 Department of Maternal-Fetal Biology, National Research Institute for Child Health and Development, Tokyo, Japan; 12 Department of Pediatrics, Hamamatsu University School of Medicine, Hamamatsu, Japan; Università degli Studi di Milano, Italy

## Abstract

**Background:**

Recent studies have revealed relative frequency and characteristic phenotype of two major causative factors for Silver-Russell syndrome (SRS), i.e. epimutation of the *H19*-differentially methylated region (DMR) and uniparental maternal disomy 7 (upd(7)mat), as well as multilocus methylation abnormalities and positive correlation between methylation index and body and placental sizes in *H19*-DMR epimutation. Furthermore, rare genomic alterations have been found in a few of patients with idiopathic SRS. Here, we performed molecular and clinical findings in 138 Japanese SRS patients, and examined these matters.

**Methodology/Principal Findings:**

We identified *H19*-DMR epimutation in cases 1–43 (group 1), upd(7)mat in cases 44–52 (group 2), and neither *H19*-DMR epimutation nor upd(7)mat in cases 53–138 (group 3). Multilocus analysis revealed hyper- or hypomethylated DMRs in 2.4% of examined DMRs in group 1; in particular, an extremely hypomethylated *ARHI*-DMR was identified in case 13. Oligonucleotide array comparative genomic hybridization identified a ∼3.86 Mb deletion at chromosome 17q24 in case 73. Epigenotype-phenotype analysis revealed that group 1 had more reduced birth length and weight, more preserved birth occipitofrontal circumference (OFC), more frequent body asymmetry and brachydactyly, and less frequent speech delay than group 2. The degree of placental hypoplasia was similar between the two groups. In group 1, the methylation index for the *H19*-DMR was positively correlated with birth length and weight, present height and weight, and placental weight, but with neither birth nor present OFC.

**Conclusions/Significance:**

The results are grossly consistent with the previously reported data, although the frequency of epimutations is lower in the Japanese SRS patients than in the Western European SRS patients. Furthermore, the results provide useful information regarding placental hypoplasia in SRS, clinical phenotypes of the hypomethylated *ARHI*-DMR, and underlying causative factors for idiopathic SRS.

## Introduction

Silver-Russell syndrome (SRS) is a rare congenital developmental disorder characterized by pre- and postnatal growth failure, relative macrocephaly, triangular face, hemihypotrophy, and fifth-finger clinodactyly [1]. Recent studies have shown that epimutation (hypomethylation) of the paternally derived differentially methylated region (DMR) in the upstream of *H19* (*H19*-DMR) on chromosome 11p15.5 and maternal uniparental disomy for chromosome 7 (upd(7)mat) account for ∼45% and 5−10% of SRS patients, respectively [1], [2]. In this regard, phenotypic assessment has suggested that birth length and weight are more reduced and characteristic body features are more frequent in patients with *H19*-DMR epimutation than in those with upd(7)mat, whereas developmental delay tends to be more frequent in patients with upd(7)mat than in those with *H19*-DMR epimutation [3], [4]. Furthermore, consistent with the notion that imprinted genes play an essential role in placental growth and development [5], placental hypoplasia has been found in both *H19*-DMR epimutation and upd(7)mat [4], [6], although comparison of placental weight has not been performed between *H19*-DMR hypomethylation and upd(7)mat. In addition, multilocus hypo- or hypermethylation and positive correlation between methylation index (MI, the ratio of methylated clones) and body and placental sizes have been reported in patients with *H19*-DMR epimutation [4], [7]–[9], and several types of rare genomic alterations have been identified in a few of SRS patients [1], [10]–[12].

Here, we report on molecular and clinical findings in 138 Japanese SRS patients, and discuss on the results obtained in this study.

## Patients and Methods

### Ethics statement

This study was approved by the Institutional Review Board Committee at the National Center for Child Health and Development. The parents of the affected children and the adult patients who can express an intention by themselves have given written informed consent to participate in this study and to publish their molecular and clinical data.

### Patients

This study consisted of 138 Japanese patients (66 males and 72 females) with SRS phenotype aged 0–30 years (median 4.1 years), including 64 previously reported patients (20 patients with variable degrees of *H19*-DMR epimutation, three patients with upd(7)mat, one patient with 46,XY/46,XY,upd(7)mat mosaicism in whom upd(7)mat cells accounted for 91–92% of leukocytes and salivary cells and for 11% of placental tissue, and 40 patients of unknown cause) [4], [6], [13]. The 138 patients had a normal karyotype in all the ≥50 lymphocytes examined, and satisfied the selection criteria proposed by Netchine et al. [14], i.e., birth length and/or birth weight ≤ –2 standard deviation score (SDS) for gestational age as a mandatory criteria plus at least three of the following five features: (i) postnatal short stature (≤ –2 SDS) at 2 year of age or at the nearest measure available, (ii) relative macrocephaly at birth, i.e., SDS for birth length or birth weight minus SDS for birth occipitofrontal circumference (OFC) ≤ –1.5, (iii) prominent forehead during early childhood, (iv) body asymmetry, and (v) feeding difficulties during early childhood. Birth and present length/height, weight, and OFC were assessed by the gestational/postnatal age- and sex-matched Japanese reference data from the Ministry of Health, Labor, and Welfare and the Ministry of Education, Science, Sports and Culture. Placental weight was assessed by the gestational age-matched Japanese reference data [15]. Clinical features were evaluated by clinicians at different hospitals who participated in this study, using the same clinical datasheet. The SRS patients were classified into three groups by the molecular studies, i.e., those with *H19*-DMR hypomethylation (epimutation) (group 1), those with upd(7)mat (group 2), and the remaining patients (group 3).

### Primers and samples

Primers utilized in this study are shown in Table S1. Leukocyte genomic DNA samples were examined in this study.

### Methylation analysis

**Figure 1:**
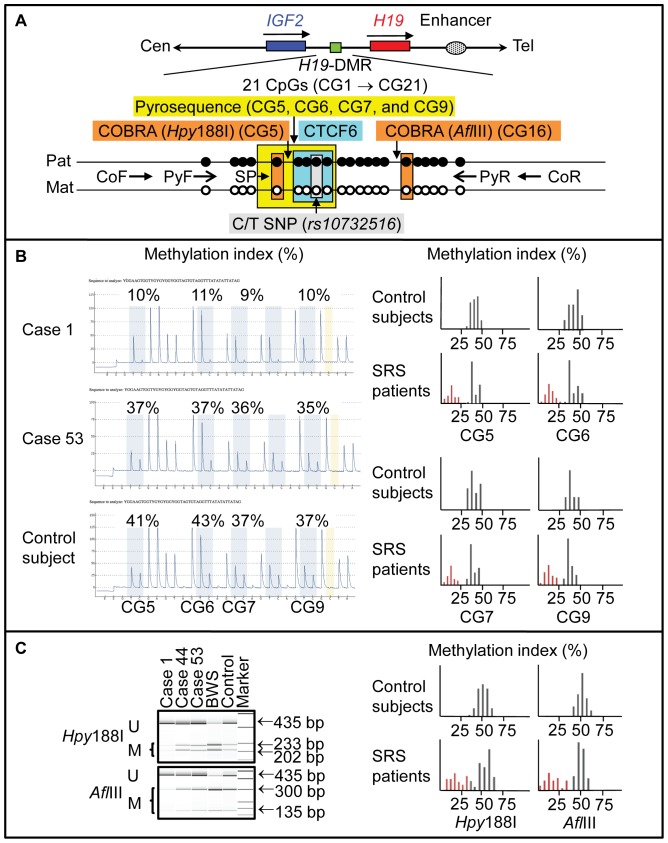
Methylation analysis of the *H19*-DMR, using bisulfite-treated genomic DNA. A. Schematic representation of a segment encompassing 21 CpG dinucleotides (CG1→CG21) within the *H19*-DMR. The cytosine residues at the CpG dinucleotides are usually methylated after paternal transmission (filled circles) and unmethylated after maternal transmission (open circles). The CTCF binding site 6 (CTCF6) is indicated with a blue rectangle; the cytosine residue at CG8 constitutes a C/T SNP (indicated with a gray rectangle). For pyrosequencing analysis, a 279 bp segment was PCR-amplified with PyF & PyR primers, and a sequence primer (SP) was hybridized to a single-stranded PCR products. Subsequently, the MIs were obtained for four CpG dinucleotides (CG5–CG7 and CG9) (indicated with a yellow rectangle). For COBRA, a 435 bp region was PCR-amplified with CoF & CoR primers, and the PCR product was digested with methylated allele-specific restriction enzymes to examine the methylation pattern of CG5 ands CG16 (the PCR products is digested with *Hpy*188I when the cytosine residue at CG5 is methylated and with *Afl*III when the cytosine residue at CG16 is methylated) (indicated with orange rectangles). *IGF2* is a paternally expressed gene, and *H19* is a maternally expressed gene. The stippled ellipse indicates the enhancer for *IGF2* and *H19*. B. Pyrosequencing data. Left part: Representative results indicating the MIs for CG5– CG7 and CG9. CG5– CG7 and CG9 are hypomethylated in case 1, and similarly methylated between case 53 and a control subject. Right part: Histograms showing the distribution of the MIs (the horizontal axis: the methylation index; and the vertical axis: the patient number). Forty-three SRS patients with low MIs are shown in red. C. COBRA data. Left part: Representative findings of PCR products loaded onto a DNA 1000 LabChip (Agilent, Santa Clara, CA, USA) after digestion with *Hpy*188I or *Afl*III. U: unmethylated clone specific bands; M: methylated clone specific bands; and BWS: Beckwith-Wiedemann syndrome patient with upd(11p15)pat. Right part: Histograms showing the distribution of the MIs.

We performed pyrosequencing analysis for the *H19*-DMR encompassing the 6th CTCF (CCCTC-binding factor) binding site that functions as the primary regulator for the monoallelic *IGF2* and *H19* expressions [16]–[18], using bisulfite treated leukocyte genomic DNA samples of all the 138 patients. The procedure was as described in the manufacturer's instructions (Qiagen, Valencia, CA, USA). In brief, a 279 bp region was PCR-amplified with a primer set (PyF and PyR) for both methylated and unmethylated clones, and a sequence primer (SP) was hybridized to a single-stranded PCR products. Subsequently, the MIs were obtained for four CpG dinucleotides (CG5–CG7 and CG9), using PyroMark Q24 (Qiagen) (the MI for CG8 was not obtained, because the “C” residue of CG8 constitutes a C/T SNP) (Figure 1A). The PyF/PyR and SP were designed by PyroMark Assay Design Software Ver2.0. While the PyF sequence contains a SNP (*rs11564736*) with a mean minor allele frequency of 5% in multiple populations, the minor allele frequency is 0% in the Japanese as well as in the Asian populations (http://browser.1000genomes.org/Homo_sapiens/Variation/Population? db = core;r = 11:2020801–2021801;v = rs11564736;vdb = variation;vf = 7864021). Thus, we utilized this PyF.

We also carried out combined bisulfite restriction analysis (COBRA) for the *H19*-DMR. The methods were as described previously [4]. In short, a 435 bp region was PCR-amplified with a primer set (CoF and CoR) that hybridize to both methylated and unmethylated clones, and MIs were obtained for two CpG dinucleotides (CG5 and CG16) after digestion of the PCR products with methylated allele-specific restriction enzymes (*Hpy*188I and *Afl*III) (Figure 1A).

Thus, we could examine CG5 by both pyrosequencing and COBRA. While we also attempted to analyze CG16 by both methods, it was impossible to design an SP for the analysis of CG16 (although we could design an SP between CG11 and CG12, clear methylation data were not obtained for CG16, probably because of the distance between the SP and CG16).

In addition, we performed COBRA for the KvDMR1 in all the 138 patients (Figure S1A) because of the possibility that epimutation of the KvDMR1 could lead to SRS phenotype via some mechanism(s) such as overexpression of a negative growth regulator *CDKN1C* [19], and for multiple DMRs on various chromosomes in patients in whom relatively large amount of DNA samples were available, as reported previously [4], [20], [21]. To define the reference ranges of MIs (minimum ∼ maximum), 50 control subjects were similarly studied with permission.

**Figure 2:**
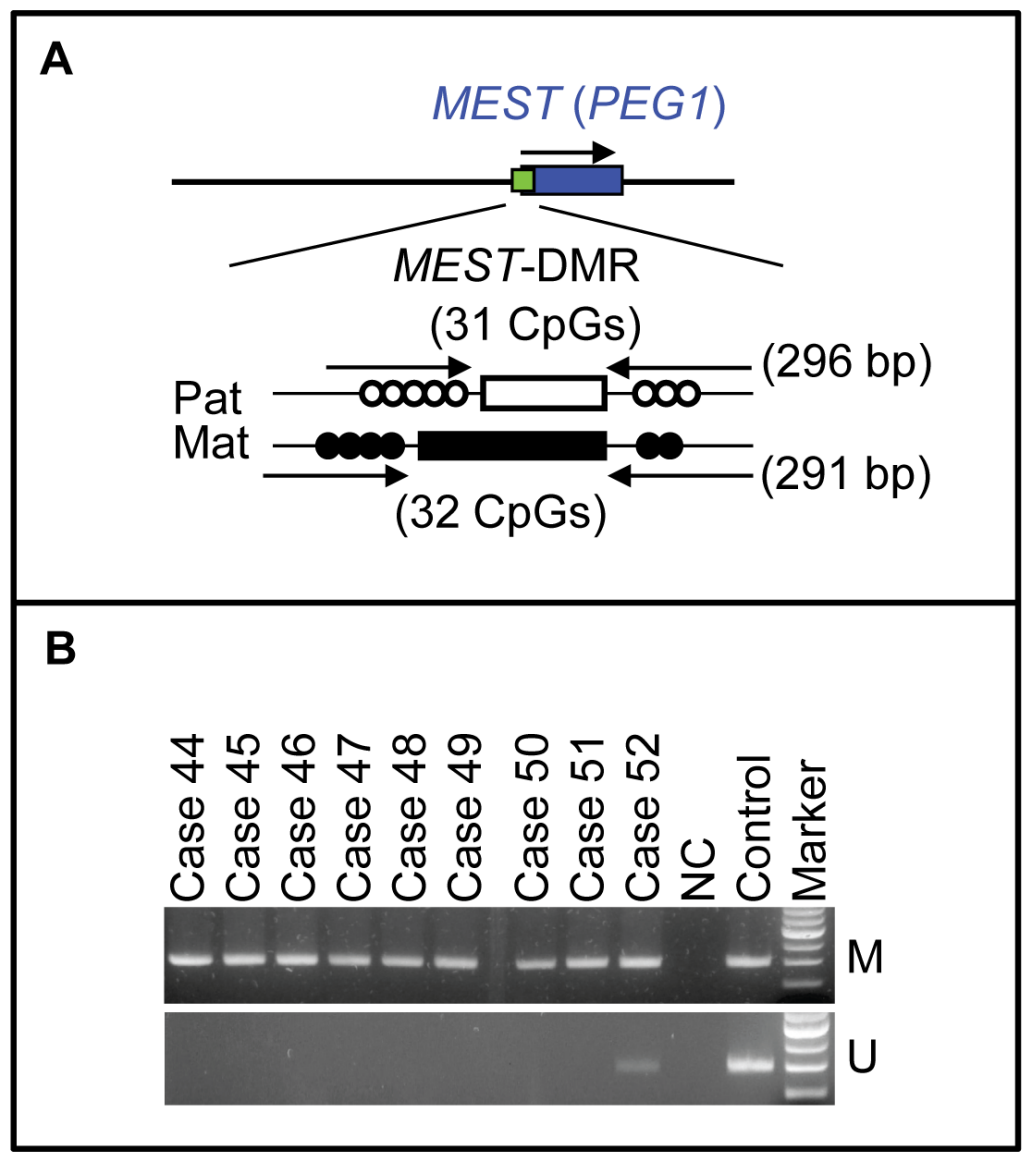
Methylated and unmethylated allele-specific PCR analysis for the *MEST*-DMR. A. Schematic representation of the *MEST*-DMR. The cytosine residues at the CpG dinucleotides are usually unmethylated after paternal transmission (open circles) and methylated after maternal transmission (filled circles). The PCR primers have been designed to hybridize either methylated or unmethylated clones. B. The results of methylation analysis with methylated and unmethylated allele-specific primers.

To screen upd(7)mat, PCR amplification was performed for the *MEST*-DMR on chromosome 7q32.2 in all the 138 patients, using methylated and unmethylated allele-specific PCR primer sets, as reported previously [6] (Figure 2A). In addition, bisulfite sequencing and direct sequencing for the primer binding sites for the *ARHI*-DMR analysis were performed in a patient (case 13) with an extremely low MI for the *ARHI*-DMR.

### Microsatellite analysis

Microsatellite analysis was performed for four loci within a ∼4.5 Mb telomeric 11p region (*D11S2071*, *D11S922*, *D11S1318*, and *D11S988*) in patients with hypomethylated *H19*-DMR, to examine the possibility of upd(11p)mat involving the *H19*-DMR. Microsatellite analysis was also carried out for nine loci widely dispersed on chromosome 7 (Table S2) in patients with abnormal methylation patterns of the *MEST*-DMR, to examine the possibility of upd(7)mat and to infer the underlying causes for upd(7)mat, i.e., trisomy rescue, gamete complementation, monosomy rescue, and post-fertilization mitotic error [22]. The methods have been reported previously [4], [6].

### Oligoarray comparative genomic hybridization (CGH)

We performed oligoarray CGH in the 138 SRS patients, using a genomewide 4×180K Agilent platform catalog array and a custom-build high density oligoarray for the 11p15.5, 7p12.2, 12q14, and 17q24 regions where rare copy number variants have been identified in several SRS patients [1], [10]–[12] as well as for the 7q32–qter region involved in the segmental upd(7)mat in four SRS patients [23]–[25]. The custom-build high density oligoarray contained 3,214 probes for 7p12.2, 434 probes for 7q32, 23,162 probes for 12q14, and 39,518 probes for 17q24, together with ∼10,000 reference probes for other chromosomal region (Agilent Technologies, Palo Alto, CA, USA). The procedure was as described in the manufacturer's instructions.

### Statistical analysis

After examining normality by χ^2^ test, the variables following the normal distribution were expressed as the mean±SD, and those not following the normal distribution were expressed with the median and range. Statistical significance of the mean was analyzed by Student's *t*-test or Welch's *t*-test after comparing the variances by *F* test, that of the median by Mann-Whitney's *U*-test, that of the frequency by Fisher's exact probability test, and that of the correlation by Pearson's correlation coefficient after confirming the normality of the variables. *P*<0.05 was considered significant.

## Results

### Identification of *H19*-DMR hypomethylation

Representative findings are shown in Figure 1B and 1C, and the MIs are summarized in Table 1. Overall, the MIs obtained by the pyrosequencing analysis tended to be lower and distributed more narrowly than those obtained by the COBRA. Despite such difference, the MIs obtained by the pyrosequencing analysis for CG5–CG7 and CG9 and by the COBRA for CG5 and CG16 were invariably below the normal range in the same 43 patients (cases 1–43) (group 1). By contrast, the MIs were almost invariably within the normal range in the remaining 95 patients, while the MIs obtained by the pyrosequencing analysis slightly (1–2%) exceeded the normal range in the same three patients (cases 136–138).

**Table 1:** The methylation indices (%) for the *H19*-DMR.

	Cases 1−43	Cases 44−138	Control subjects
Pyrosequencing analysis			
CG5	4–24	35−50	33−48
CG6	5–26	36−53	34−51
CG7	4–24	35−49	30−47
CG9	5–23	34−48	30−46
COBRA			
CG5 (*Hpy*188I)	3.3−35.1	37.8−60.8	36.2−58.5
CG16 (*Afl*III)	4.1−35.0	43.0−59.4	38.7−60.0

The position of examined CpG dinucleotides (CG5–7, CG9, and CG16) is shown in Figure 1A.

COBRA: combined bisulfite restriction analysis.

In the 43 cases of group 1, microsatellite analysis for four loci at the telomeric 11p region excluded maternal upd in 14 cases in whom parental DNA samples were available; in the remaining 29 cases, microsatellite analysis identified two alleles for at least one locus, excluding maternal uniparental isodisomy for this region. Furthermore, oligoarray CGH for the chromosome 11p15.5 region showed no copy number alteration such as duplication of maternally derived *H19*-DMR and deletion of paternally derived *H19*-DMR. For the KvDMR1, the MIs of the 138 patients remained within the reference range (Fig. S1B and C).

### Identification of upd(7)mat

Methylation analysis for the *MEST*-DMR revealed that unmethylated bands were absent from eight patients and remained faint in a single patient (cases 44–52) (group 2) (Figure 2B). Subsequent microsatellite analysis confirmed upd(7)mat in the eight patients and mosaic upd(7)mat in the remaining one patient, and indicated trisomy rescue or gamete complementation type upd(7)mat in cases 44–48, monosomy rescue or post-fertilization mitotic error type upd(7)mat in cases 49–51, and post-fertilization mitotic error type mosaic upd(7)mat in case 52 (Table S2).

### Multiple DMR analysis

**Figure 3:**
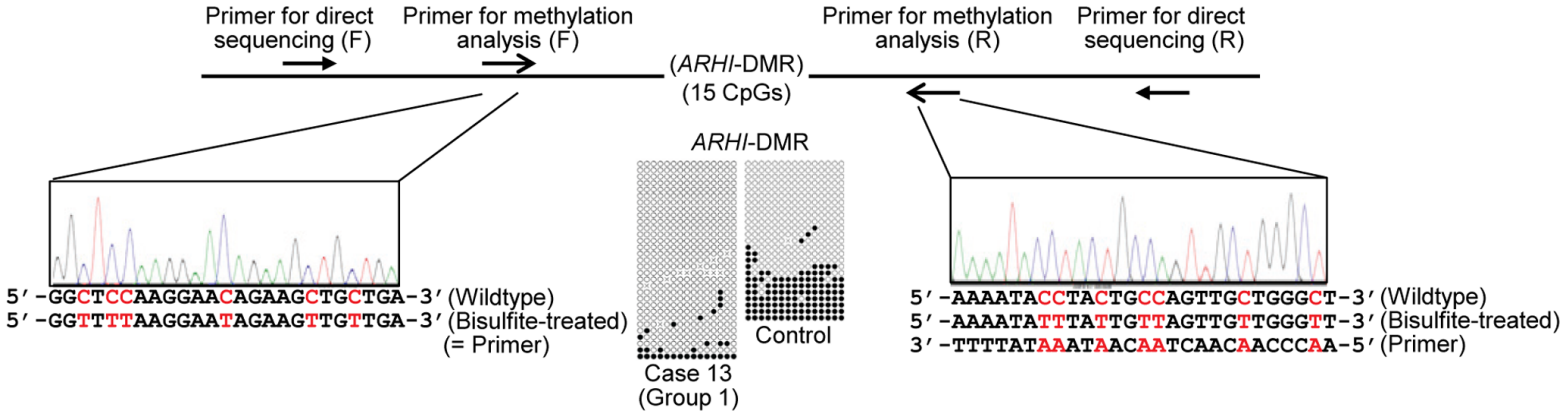
Analysis of the *ARHI*-DMR in case 13. For bisulfite sequencing, each line indicates a single clone, and each circle denotes a CpG dinucleotide; the cytosine residues at the CpG dinucleotides are usually unmethylated after paternal transmission (open circles) and methylated after maternal transmission (filled circles). Electrochromatograms delineate the sequences of the primer binding sites utilized for the methylation analysis.

We examined 17 autosomal DMRs other than the *H19*-DMR in 14 patients in group 1, four patients in group 2, and 20 patients in group 3, and the *XIST*-DMR in eight female patients in group 1, one female patient in group 2, and five female patients in group 3 (Table S3). The MIs outside the reference ranges were identified in five of 14 examined cases (35.7%) and six of a total of 246 examined DMRs (2.4%) in group 1. In particular, a single case with the mean MI value of 23 obtained by the pyrosequencing analysis for CG5–CG7 and CG9 had an extremely low MI for the *ARHI*-DMR (case 13 of group 1). This extreme hypomethylation was confirmed by bisulfite sequencing, and direct sequencing showed normal sequences of the primer-binding sites, thereby excluding the possibility that such an extremely low MI could be due to insufficient primer hybridization because of the presence of a nucleotide variation within the primer-binding sites (Figure 3). Furthermore, no copy number variation involving the *ARHI*-DMR was identified by CGH analysis using a genomewide catalog array. Consistent with upd(7)mat, three DMRs on chromosome 7 were extremely hypermethylated in four examined cases of group 2. Only a single DMR was mildly hypermethylated in a total of 345 examined DMRs in group 3. The abnormal MIs, except for those for the *H19*-DMR in group 1 and for the three DMRs on chromosome 7 in group 2, were confirmed by three times experiments.

### Oligonucleotide array CGH

**Figure 4:**
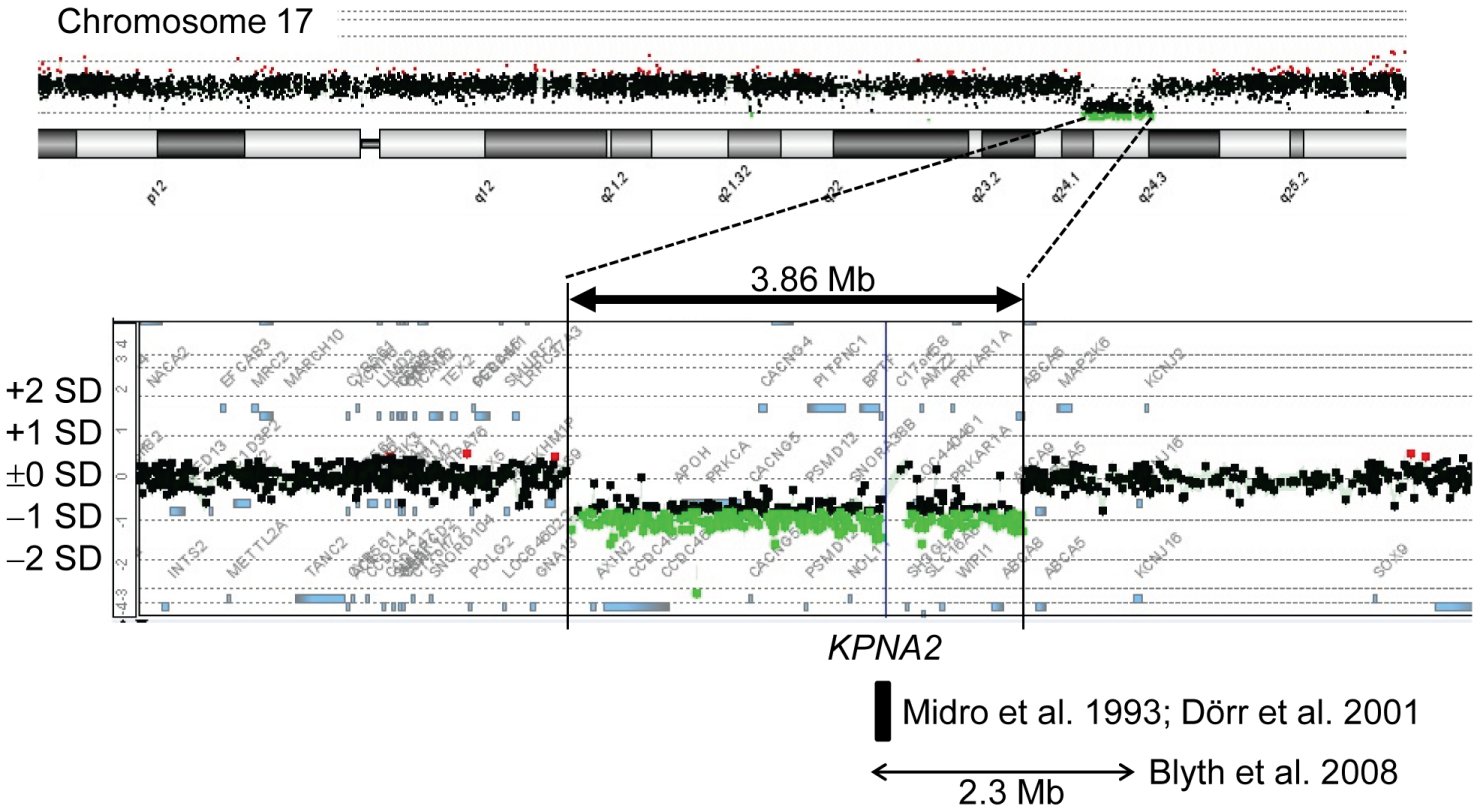
Oligonucleotide array CGH in case 73, showing a ∼3.86 Mb deletion at chromosome 17q24. The black, the red, and the green dots denote signals indicative of the normal, the increased(>+0.5), and the decreased (< –1.0) copy numbers, respectively. The horizontal bar with arrowheads indicates a ∼2.3 Mb deletion identified in a patient with Carney complex and SRS-like phenotype [44], and the black square represent a ∼65 kb segment harboring the breakpoint of a *de novo* translocation 46,XY,t(1;17)(q24;q23–q24) identified in a patient with SRS phenotype [45], [46].

A ∼3.86 Mb deletion at chromosome 17q24 was identified in a single patient (case 73 of group 3) (Figure 4).

### Epigenotype-phenotype analysis

Clinical findings of SRS patients in groups 1–3 are summarized in Table 2. All the patients met the mandatory criteria, and most patients in each group had severely reduced birth length and weight (both≤–2 SDS). For the five clinical features utilized as scoring system criteria, while 23.2% of patients in group 1 and 22.2% of patients in group 2 exhibited all the five features, there was no patient in group 3 who was positive for all the five features. By contrast, while 39.5% of patients in group 1 and 33.3% of patients in group 2 manifested just three of the five features, 77.6% of patients in group 3 were positive for just three features. In particular, the frequencies of relative macrocephaly at birth and body asymmetry were low in group 3, while those of the remaining three scoring system criteria including prominent forehead during early childhood were similar among groups 1–3.

**Table 2:** Phenotypic comparison in three groups of patients with Silver-Russell syndrome.

	*H19*-DMR hypomethylation	Upd(7)mat	Unknown	*P*-value		
	(Group 1)	(Group 2)	(Group 3)	G1 vs. G2	G1 vs. G3	G2 vs. G3
Patient number	43 (31.2%)	9 (6.5%)	85 (62.0%)			
Mandatory criteria	43/43 (100%)	9/9 (100%)	85/85 (100%)	1.000	1.000	1.000
Scoring system criteria (5/5)	10/43 (23.2%)	2/9 (22.2%)	0/85 (0.00%)	0.965	**1.52×10^−4^**	**2.58×10^−2^**
Scoring system criteria (4/5)	16/43 (37.2%)	4/9 (44.4%)	19/85 (22.4%)	0.792	**1.45×10^−2^**	0.145
Scoring system criteria (3/5)	17/43 (39.5%)	3/9 (33.3%)	66/85 (77.6%)	0.821	**7.17×10^−4^**	0.161
Gestational age (weeks:days)	38∶0 (34∶3∼40∶0) (n = 36)	38∶0 (34∶4∼40∶0) (n = 9)	37∶6 (27∶1∼41∶4) (n = 65)	0.877	0.120	0.450
BL (SDS)	–4.13±2.01 (n = 31)	–3.18±1.16 (n = 9)	–2.93±1.43 (n = 60)	**2.67×10^−2^**	**6.69×10^−5^**	0.619
BW (SDS)	–3.50±0.85 (n = 42)	–2.90±0.64 (n = 9)	–2.71±1.14 (n = 64)	**3.28×10^−2^**	**5.87×10^−4^**	0.640
BL≤–2 SDS and/or BW≤–2 SDS*	43/43 (100%)	9/9 (100%)	85/85 (100%)	1.000	1.000	1.000
BL≤–2 SDS and BW≤–2 SDS	39/43 (90.7%)	7/9 (77.8%)	76/85 (89.4%)	0.474	0.821	0.304
BOFC (SDS)	–0.54±1.22 (n = 29)	–1.44±0.47 (n = 9)	–1.92±1.09 (n = 48)	**3.74×10^−2^**	**1.52×10^−6^**	0.202
BL (SDS) – BOFC (SDS)	–3.70±2.02 (n = 27)	–1.73±1.20 (n = 9)	–0.943±1.48 (n = 43)	**1.02×10^−2^**	**3.40×10^−9^**	0.111
BW (SDS) – BOFC (SDS)	–3.21±1.20 (n = 27)	–1.53±0.57 (n = 9)	–1.04±1.55 (n = 48)	0.326	**7.38×10^−9^**	0.331
Relative macrocephaly at birth†BL or BW (SDS) – BOFC (SDS)≤–1.5	29/29 (100%)	7/9 (77.8%)	16/45 (35.6%)	0.341	**3.67×10^−8^**	**2.05×10^−2^**
Present age (years:months)	4.1 (0∶6∼30∶6) (n = 31)	4.8 (2∶4∼25∶2) (n = 9)	4.3 (0∶1∼18∶6) (n = 60)	0.437	0.813	0.335
PH (SDS)	–3.58±1.65 (n = 35)	–3.77±1.13 (n = 9)	–3.17±1.50 (n = 61)	0.757	0.218	0.253
PH≤–2 SDS (≥2 years)†	29/35 (82.5%)	8/9 (88.9%)	52/61 (85.2%)	0.760	0.758	0.772
PW (SDS)	–3.15±1.16 (n = 32)	–2.77±0.76 (n = 9)	–2.77±1.34 (n = 59)	0.362	0.144	0.968
POFC (SDS)	–1.16±1.18 (n = 21)	–0.01±0.91 (n = 9)	–1.81±1.57 (n = 35)	**2.01×10^−3^**	0.107	**3.08×10^−3^**
PH (SDS) – POFC (SDS)	–2.47±1.63 (n = 16)	–3.62±1.38 (n = 8)	–1.55±1.82 (n = 35)	0.103	**4.39×10^−2^**	**1.64×10^−2^**
PW (SDS) – POFC (SDS)	–2.84±1.31 (n = 21)	–2.69±1.36 (n = 9)	–1.08±1.71 (n = 35)	0.782	**2.54×10^−2^**	**1.90×10^−4^**
Relative macrocephaly at presentPH or PW (SDS) – POFC (SDS)≤–1.5	20/21 (95.2%)	8/8 (100%)	29/43 (67.4%)	0.223	**4.77×10^−3^**	0.156
Triangular face during early childhood	42/43 (97.7%)	8/9 (88.9%)	65/65 (100%)	0.442	0.0773	**5.98×10^−3^**
Prominent forehead during early childhood†	31/37 (83.8%)	7/9 (100%)	41/53 (77.4%)	0.200	0.456	0.978
Ear anomalies	14/35 (40.0%)	3/9 (33.3%)	15/55 (27.3%)	0.717	0.290	0.823
Irregular teeth	12/26 (46.2%)	4/9 (44.4%)	12/45 (26.7%)	0.930	0.0968	0.291
Body asymmetry†	30/37 (81.1%)	3/9 (33.3%)	19/59 (32.2%)	**4.77×10^−3^**	**3.51×10^−6^**	0.947
Clinodactyly	29/37 (78.4%)	5/9 (55.6%)	50/58 (86.2%)	0.167	0.323	**2.68×10^−2^**
Brachydactyly	30/38 (78.9%)	2/9 (22.2%)	34/56 (60.7%)	1.16×10**^−^** ^3^	0.0642	**3.24×10^−2^**
Syndactyly	3/36 (8.3%)	0/9 (0.00%)	3/52 (5.77%)	0.375	0.641	0.464
Simian crease	4/26 (15.4%)	2/7 (28.6%)	6/49 (12.2%)	0.429	0.705	0.252
Muscular hypotonia	17/32 (53.1%)	5/9 (55.6%)	12/50 (24.0%)	0.898	**7.49×10^−3^**	0.0564
Developmental delay	18/37 (48.6%)	6/9 (66.7%)	25/54 (46.3%)	0.337	0.826	0.262
Speech delay	8/31 (25.8%)	6/9 (66.7%)	18/43 (41.9%)	**2.55×10^−2^**	0.156	0.179
Feeding difficulty†	16/34 (47.1%)	6/9 (66.7%)	25/51 (49.0%)	0.301	0.860	0.333
Placental weight (SDS)	–2.10±0.74 (n = 14)	–1.72 ± 0.74 (n = 6)^a^	–1.02±0.86 (n = 18)	0.312	**4.12×10^−3^**	**8.24×10^−3^**
Paternal age at childbirth (years:months)	32∶0 (19∶0∼52∶0) (n = 24)	35∶0 (27∶0∼48∶0) (n = 9)	32∶0 (25∶0∼46∶0) (n = 45)	0.223	1.00	0.105
Maternal age at childbirth (years:months)	32∶0 (19∶0∼43∶0) (n = 25)	33∶0 (25∶0∼42∶0) (n = 9)^b^	30∶0 (22∶0∼43∶0) (n = 46)	0.275	0.765	0.117

BL: birth length; BW: birth weight; BOFC: birth occipitofrontal circumference; PH: present height; PW: present weight; POFC: present occipitofrontal circumference, and SDS: standard deviation score.

For body features, the denominators indicate the number of patients examined for the presence or absence of each feature, and the numerators represent the number of patients assessed to be positive for that feature.

* Mandatory criteria and †five clinical features utilized as selection criteria for Silver-Russell syndrome proposed by Netchine et al. [14].

Significant *P*-values(<0.05) are boldfaced.

a Placental weight SDS is –1.68, –2.55, –2.24, –1.12, –2.14 and –0.60 in case 46, 47, 49, 50, 51 and 52, respectively; the placental weight SDS is –1.95±0.57 in five cases except for case 52 with mosaic upd(7)mat.

b Maternal childbearing age is 32, 32, 33, 42, 32, 34, 33, 25 and 36 years in case 44–52, respectively.

Phenotypic comparison between groups 1 and 2 revealed that birth length and weight were more reduced and birth OFC was more preserved in group 1 than in group 2, despite comparable gestational age. In the postnatal life, present height and weight became similar between the two groups, whereas present OFC became significantly smaller in group 1 than in group 2. Body asymmetry and brachydactyly were more frequent and speech delay was less frequent in group 1 than in group 2. Placental weight was similar between the two groups, and became more similar after excluding case 52 with mosaic upd(7)mat (see legends for Table 2). Parental age at childbirth was also similar between the two groups. In group 2, placental weight was grossly similar among examined cases, as was parental age at childbirth (see legends for Table 2).

Case 13 with an extremely low MI for the *ARHI*-DMR and case 73 with a cryptic deletion at chromosome 17q24 had no specific phenotype other than SRS-like phenotype (Table S4). However, of the five clinical features utilized as scoring system criteria, all the five features were exhibited by case 13 and just three features were manifested by case 73. In addition, cases 136–138 with slightly elevated MIs for CG5–CG7 and CG9, and cases with multilocus methylation abnormalities, had no particular phenotype other than SRS-compatible clinical features.

### Correlation analysis

In group 1, the mean value of the MIs for CG5–CG7 and CG9 obtained by pyrosequencing analysis was positively correlated with the birth length and weight, the present height and weight, and the placental weight, but with neither the birth nor the present OFC (Table 3). Such correlations with the growth parameters were grossly similar but somewhat different for the MIs obtained by COBRA (Table S5). Furthermore, the placental weight was positively correlated with the birth weight and length, but not with the birth OFC. Such positive correlations were not found in groups 2 and 3.

**Table 3:** Correlation analyses in patients with *H19*-DMR hypomethylations.

Parameter 1		Parameter 2	*r*	*P*-value
Methylation index (%)*	vs.	Birth length (SDS)	0.647	**6.70×10^−3^**
		Birth weight (SDS)	0.590	**7.80×10^−3^**
		Birth OFC (SDS)	0.190	0.498
		Present height (SDS)	0.612	**5.33×10^−3^**
		Present weight (SDS)	0.605	**7.81×10^−3^**
		Present OFC (SDS)	–0.166	0.647
		Placental weight (SDS)	0.809	**8.30×10^−3^**
Placental weight (SDS)	vs.	Birth weight (SDS)	0.717	**8.64×10^−3^**
		Birth length (SDS)	0.636	**2.63×10^−2^**
		Birth OFC (SDS)	0.400	0.198

SDS: standard deviation score; and OFC: occipitofrontal circumference.

* The mean value of MIs for CG5, CG6, CG7, and CG9 obtained by pyrosequencing analysis.

Significant *P*-values(<0.05) are boldfaced.

## Discussion

The present study identified hypomethylation of the *H19*-DMR and upd(7)mat in 31.2% and 6.5% of 138 Japanese SRS patients, respectively. In this regard, the normal KvDMR1 methylation patterns indicate that the aberrant methylation in 43 cases of group 1 is confined to the *H19*-DMR. Furthermore, oligoarray CGH excludes copy number variants involving the *H19*-DMR, and microsatellite analysis argues against segmental maternal isodisomy that could be produced by post-fertilization mitotic error [26]. These findings imply that the *H19*-DMR hypomethylation is due to epimutation (hypomethylation of the normally methylated *H19*-DMR of paternal origin).

The frequency of epimutations detected in this study is lower than that reported in Western European SRS patients [1], [2], [14], although the frequency of upd(7)mat is grossly similar between the two populations [2], [11], [14], [27], [28]. In this context, it is noteworthy that, of the five scoring system criteria, the frequencies of relative macrocephaly at birth and body asymmetry were low in group 3, while those of the remaining three scoring system criteria were similar among groups 1–3. Since relative macrocephaly and body asymmetry are characteristic of *H19*-DMR epimutation, the lack of these two features in a substantial fraction of cases in group 3 would primarily explain the low frequency of *H19*-DMR epimutations in this study. In group 3, furthermore, the low prevalence of relative macrocephaly at birth appears to be discordant with the high prevalence of prominent forehead during early childhood. Since relative macrocephaly was evaluated by an objective method (SDS for birth length or birth weight minus SDS for birth OFC≤–1.5) and prominent forehead was assessed by a subjective impression of different clinicians, it is recommended to utilize relative macrocephaly as a more important and reliable feature in the scoring system than prominent forehead. In addition, the difference in the ethnic group might also be relevant to the low frequency of *H19*-DMR epimutations in this study.

Epigenotype-phenotype correlations in this study are grossly similar to those previously reported in Western European SRS patients [1]–[3]. Cases 1–43 in group 1 with *H19*-DMR epimutation had more reduced birth weight and length, more preserved birth OFC and more reduced present OFC, more frequent body features, and less frequent speech delay than case 44–52 in group 2 with upd(7)mat, although the difference in the prevalence of somatic features appears to be less remarkable in this study than in the previous studies [3], [4]. This provides further support for the presence of relatively characteristic clinical features in *H19*-DMR epimutation and upd(7)mat [1]–[3]. In this context, previous studies have indicated biallelic *IGF2* expression in the human fetal choroid plexus, cerebellum, and brain, and monoallelic *IGF2* expression in the adult brain, while the precise brain tissue(s) with such a unique expression pattern remains to be clarified [29], [30], [31]. This may explain why the birth OFC is well preserved and the present OFC is reduced in group 1. However, since the difference in present OFC between groups 1 and 2 is not necessarily significant in the previous studies [32], the postnatal OFC growth awaits further investigations.

Placental weight was similarly reduced in groups 1 and 2. Thus, placental weight is unlikely to represent an indicator for the discrimination between the two groups, although the present data provide further support for imprinted genes being involved in placental growth, with growth-promoting effects of *PEGs* and growth-suppressing effects of *MEGs* [5], [6]. It should be pointed out, however, that the placental hypoplasia could be due to some other genetic or environmental factor(s). In particular, while placental weight was apparently similar among cases of group 2, possible confined placental mosaicism [33], [34] with trisomy for chromosome 7 may have exerted some effects on placental growth in cases with trisomy rescue type upd(7)mat.

Correlation analysis would imply that the *IGF2* expression level, as reflected by the MI of the *H19*-DMR, plays a critical role in the determination of pre- and postnatal body (stature and weight) and placental growth in patients with *H19*-DMR epimutation. Since the placental weight was positively correlated with the birth length and weight, the reduced *IGF2* expression level appears to have a similar effect on the body and the placental growth. Furthermore, the lack of correlations between the MI and birth and present OFC and between placental weight and birth OFC would be compatible with the above mentioned *IGF2* expression pattern in the central nervous system [29]. Although the MI would also reflect the *H19* expression level, this would not have a major growth effect. It has been implicated that *H19* functions as a tumor suppressor [35], [36].

Multilocus analysis revealed co-existing hyper- and hypomethylated DMRs predominantly in cases of group 1, with frequencies of 35.7% of examined patients and 2.4% of examined DMRs. The results are grossly consistent with the previous data indicating that co-existing abnormal methylation patterns of DMRs are almost exclusively identified in patients with *H19*-DMR epimutation with frequencies of 9.5∼30.0% of analyzed patients and 1.8∼5.2% of a total of analyzed DMRs [7]–[9]. Notably, the co-existing methylation abnormalities were predominantly observed as mild hypermethylations of maternally methylated DMRs and were restricted to a single DMR or two DMRs in patients with multilocus abnormalities. Such findings are obviously inexplicable not only by assuming a *ZFP57* mutation that is known to cause severely abnormal methylation patterns of multiple DMRs or a *ZAC1* mutation that may affect methylation patterns of multiple DMRs [37]–[39], but also by assuming defective maintenance of methylation in the postzygotic period [7]. Thus, some factor(s) susceptible to the co-occurrence of hypomethylation of the *H19*-DMR and hypermethylation of other DMR(s) might be operating during a gametogenic or postzygotic period in cases with *H19*-DMR epimutation.

The patients with multilocus methylation abnormalities had no specific clinical features other than SRS-compatible phenotype. Previous studies have also indicated grossly similar SRS-like phenotype between patients with monolocus and multilocus hypomethylations [7], although patients with multilocus hypomethylation occasionally have apparently severe clinical phenotype [7]. These findings would argue for the notion that the *H19*-DMR epimutation has an (epi)dominant clinical effect. Indeed, *H19*-DMR hypomethylation has led to SRS-like phenotype in a patient with parthenogenetic chimerism/mosaicism [21], whereas *H19*-DMR hypermethylation has resulted in Beckwith-Wiedemann syndrome-like phenotype in patients with androgenetic mosaicism [40].

An extremely hypomethylated *ARHI*-DMR was found in case 13. In this regard, it is known that *ARHI* with a potentially cell growth suppressor function is normally expressed from paternally inherited chromosome with unmethylated *ARHI*-DMR [41]. Indeed, hypermethylation of the *ARHI*-DMR, which is predicted to result in reduced expression of *ARHI*, has been identified as a tumorigenic factor for several cancers with an enhanced cell growth function [42], [43]. Thus, it is possible that hypomethylation of the *ARHI*-DMR has led to overexpression of *ARHI*, contributing to the development of typical SRS phenotype in the presence of a low but relatively preserved MI of the *H19*-DMR in case 13.

Oligonucleotide array CGH identified a ∼3.86 Mb deletion at chromosome 17q24 in case 73 of group 3. This provides further support for the presence of rare copy number variants in several SRS patients and the relevance of non-imprinted gene(s) to the development of SRS [10]. Interestingly, the microdeletion overlap with that identified in a patient with Carney complex and SRS-like features [44], and the overlapping region encompasses a ∼65 kb segment defining the breakpoint of a *de novo* reciprocal translocation involving 17q23–q24 in a patient with SRS-like phenotype (Figure 4) [45], [46]. Furthermore, the translocation breakage has affected *KPNA2* involved in the nuclear transport of proteins [46]–[48]. Thus, *KPNA2* has been regarded as a candidate gene for SRS, although mutation analysis of *KPNA2* has failed to detect a disease-causing mutation in SRS patients [49].

Lastly, it would be worth discussing on the comparison between pyrosequencing analysis and COBRA. Since the same 43 patients were found to have low MIs by both analyses, this implies that both methods can be utilized as a diagnostic tool. While the distribution of the MIs was somewhat different between the two methods, this would primarily be due to the difference in the employed methods such as the hybridization efficiency of utilized primers. Importantly, pyrosequencing analysis was capable of studying plural CpG dinucleotides at the CTCF6 binding site, whereas COBRA examined only single CpG dinucleotides outside the CTCF6 binding site. Thus, the MIs obtained by pyrosequencing analysis would be more accurate than those obtained by COBRA in terms of *IGF2* expression levels, and this would underlie the reasonable correlations of MIs yielded by pyrosequencing analysis with body and placental growth parameters.

In summary, the present study provides useful information for the definition of molecular and clinical findings in SRS. However, several matters still remain to be elucidated, including underlying mechanisms in SRS patients with no *H19*-DMR epimutation or upd(7)mat and the DMR(s) and imprinted gene(s) responsible for the development of SRS in patients with upd(7)mat. Furthermore, while advanced maternal age at childbirth has been shown to be a predisposing factor for the development of upd(15)mat because of increased non-disjunction at meiosis 1 [50], such studies remain fragmentary for upd(7)mat, primarily because of the relative paucity of upd(7)mat. Further studies will permit a better characterization of SRS.

## Supporting Information

Figure S1 Methylation analysis of the KvDMR1 using COBRA. A. Schematic representation of the KvDMR1. A 326 bp region harboring 24 CpG dinucleotides was studied. The cytosine residues at the CpG dinucleotides are usually methylated after paternal transmission (filled circles) and unmethylated after maternal transmission (open circles); after bisulfite treatment, this region is digested with *Hpy*188I when the cytosine at the 5th CpG dinucleotide (indicated with a green rectangle) is methylated and with *Eci*I when the cytosines at the 22nd CpG dinucleotide (indicated with a pink rectangle) is methylated. *KCNQ1OT1* is a paternally expressed gene, and *KCNQ1* and *CDKN1C* are maternally expressed genes. B. Representative COBRA results. U: unmethylated clone specific bands; M: methylated clone specific bands; and BWS: Beckwith-Wiedemann syndrome patient with upd(11p15)pat. C. Histograms showing the distribution of the MIs (the horizontal axis: the methylation index; and the vertical axis: the patient number).
(TIF)

Table S1 Primers utilized in the methylation analysis and microsatellite analysis.
(XLS)

Table S2 The results of microsatellite analysis.
(XLSX)

Table S3 Methylation indices for multiple differetially methylated resions (DMRs) obtained by COBRA in 38 patients with Silver-Russell syndrome.
(XLSX)

Table S4 Clinical findings in two unique patients.
(DOC)

Table S5 Correlation analyses in patients with H19-DMR hypomethylations.
(DOC)
