# Supplementary figures and images for: Molecular and Clinical Studies in 138 Japanese Patients with Silver-Russell Syndrome
Source: PLoS One. 2013 Mar 22;8(3):e60105. doi: 10.1371/journal.pone.0060105 (PMC3606247; doi:10.1371/journal.pone.0060105)

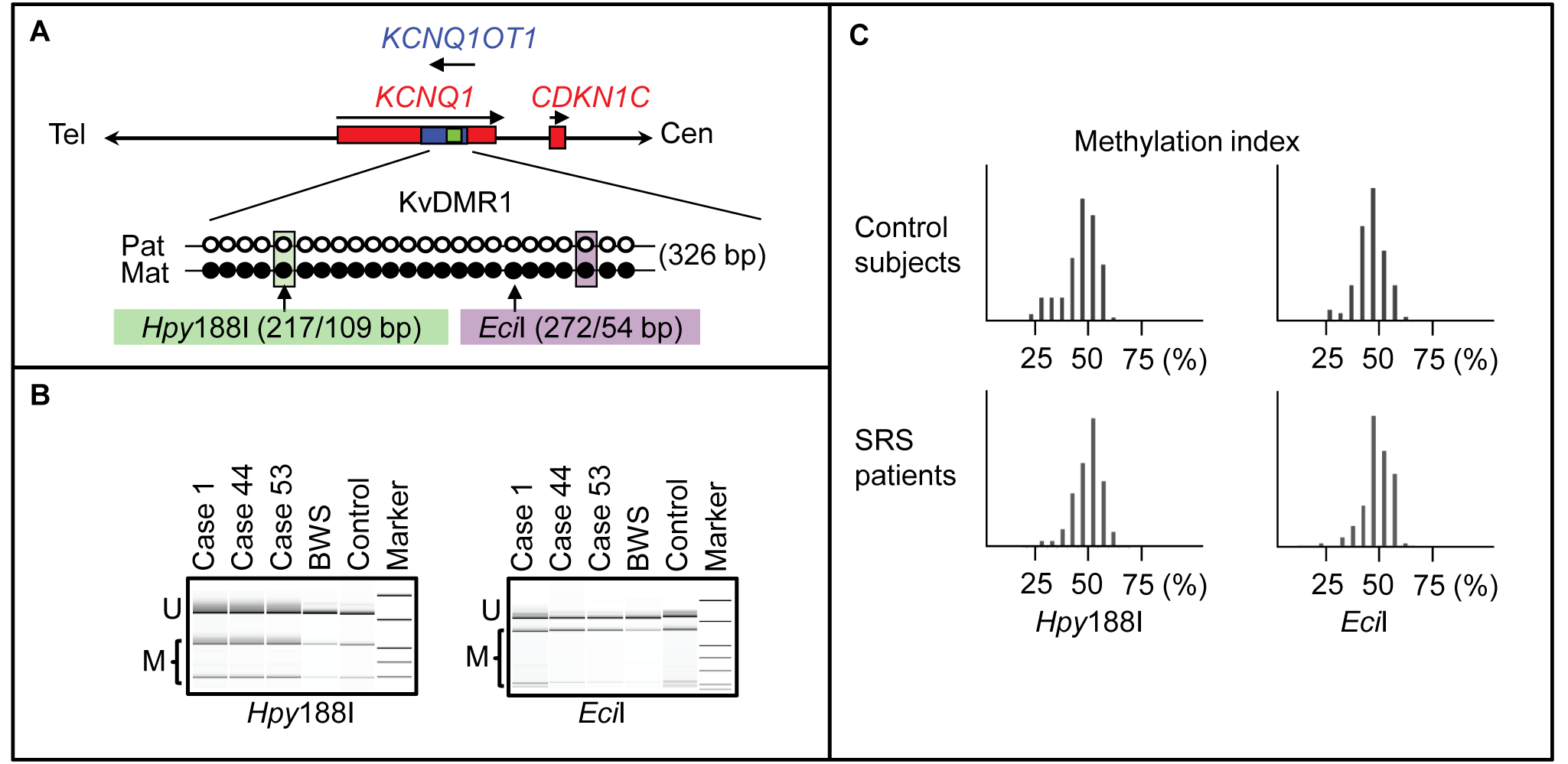

Supplement: Figure S1 — Methylation analysis of the KvDMR1 using COBRA. A. Schematic representation of the KvDMR1. A 326 bp region harboring 24 CpG dinucleotides was studied. The cytosine residues at the CpG dinucleotides are usually methylated after paternal transmission (filled circles) and unmethylated after maternal transmission (open circles); after bisulfite treatment, this region is digested with Hpy188I when the cytosine at the 5th CpG dinucleotide (indicated with a green rectangle) is methylated and with EciI when the cytosines at the 22nd CpG dinucleotide (indicated with a pink rectangle) is methylated. KCNQ1OT1 is a paternally expressed gene, and KCNQ1 and CDKN1C are maternally expressed genes. B. Representative COBRA results. U: unmethylated clone specific bands; M: methylated clone specific bands; and BWS: Beckwith-Wiedemann syndrome patient with upd(11p15)pat. C. Histograms showing the distribution of the MIs (the horizontal axis: the methylation index; and the vertical axis: the patient number). (TIF) [file pone.0060105.s001.tif]
