# Supplementary material for: Molecular and Clinical Studies in 138 Japanese Patients with Silver-Russell Syndrome
Source: PLoS One. 2013 Mar 22;8(3):e60105. doi: 10.1371/journal.pone.0060105 (PMC3606247; doi:10.1371/journal.pone.0060105)
Supplement: Table S4 — Clinical findings in two unique patients. (DOC) [file pone.0060105.s005.doc]

| **Table S4.** Clinical findings in two unique patients. | | |
| --- | --- | --- |
|  | Case 13 | Case 73 |
|  | *H19*-DMR &  *ARHI*-DMR hypomethylations | Chromosome 17q24 microdeletion |
| Gestational age (weeks:days) | 37:3 | 34:0 |
| Present age (years:months) | 1:4 | 2:0 |
| BL (SDS) | −5.6 | −1.7 |
| BW (SDS) | −3.6 | −3.1 |
| BL ≤ –2 SDS and/or BW ≤ –2 SDS* | + | + |
| BOFC (SDS) | −1.5 | −2.5 |
| Relative macrocephaly at birth†  BL or BW (SDS) – BOFC (SDS) ≤ –1.5 | + | − |
| PH（SDS） | −3.3 | −5.2 |
| PW (SDS) | −3.3 | −3.6 |
| PH ≤ –2 SDS (≥2 years)† | + | + |
| POFC (SDS) | −1.5 | −1.7 |
| Relative macrocephaly at present  PH or PW (SDS) – POFC (SDS) ≤ –1.5 | + | + |
| Triangular face during early childhood | + | + |
| Prominent forehead during early childhood† | + | + |
| Ear anomalies | + | + |
| Irregular teeth | + | + |
| Body asymmetry† | + | + |
| Clinodactyly | + | − |
| Brachydactyly | + | + |
| Syndactyly | − | + |
| Simian crease | − | − |
| Muscular hypotonia | − | + |
| Developmental delay | + | + |
| Speech delay | − | − |
| Feeding difficulties† | + | − |
| Placental weight (SDS) | −1.7 | −1.7 |
| Paternal age at childbirth (years:months) | Unknown | 32:6 |
| Maternal age at childbirth (years:months) | 37:2 | 25:5 |
| BL: birth length; BW: birth weight; BOFC: birth occipitofrontal circumference; PH: present height; PW: present weight; POFC: present occipitofrontal circumference, and SDS: standard deviation score.  *Mandatory criteria and †five clinical features utilized as selection criteria for Silver-Russell syndrome proposed by Netchine et al. [14]. | | |
