# Supplementary material for: Molecular and Clinical Studies in 138 Japanese Patients with Silver-Russell Syndrome
Source: PLoS One. 2013 Mar 22;8(3):e60105. doi: 10.1371/journal.pone.0060105 (PMC3606247; doi:10.1371/journal.pone.0060105)
Supplement: Table S5 — Correlation analyses in patients with H19-DMR hypomethylations. (DOC) [file pone.0060105.s006.doc]

| **Table S5.** Correlation analyses in patients with *H19*-DMR hypomethylations. | | | | |
| --- | --- | --- | --- | --- |
| Parameter 1 |  | Parameter 2 | *r* | *P*-value |
| Methylation index (%) | vs. | Birth length (SDS) | 0.335 | 0.174 |
| CG5 (COBRA-*Hpy*188I) |  | Birth weight (SDS) | 0.514 | **1.73×10^-2^** |
|  |  | Birth OFC (SDS) | 0.372 | 0.128 |
|  |  | Present height (SDS) | 0.319 | 0.159 |
|  |  | Present weight (SDS) | 0.551 | **1.18×10^-2^** |
|  |  | Present OFC (SDS) | 0.620 | **1.81×10^-2^** |
|  |  | Placental weight (SDS) | 0.673 | **2.32×10^-2^** |
| Methylation index (%) | vs. | Birth length (SDS) | 0.290 | 0.242 |
| CG16 (COBRA-*Afl*III) |  | Birth weight (SDS) | 0.572 | **6.70×10^-3^** |
|  |  | Birth OFC (SDS) | 0.264 | 0.289 |
|  |  | Present height (SDS) | 0.507 | **1.89×10^-2^** |
|  |  | Present weight (SDS) | 0.604 | **4.79×10^-3^** |
|  |  | Present OFC (SDS) | 0.656 | **1.09×10^-2^** |
|  |  | Placental weight (SDS) | 0.800 | **3.13×10^-3^** |
| COBRA: combined bisulfite restriction analysis; SDS, standard deviation score; and OFC, occipitofrontal circumference.  Significant *P*-values (< 0.05) are boldfaced. | | | | |
